# Development of three-dimensional forward modeling and observation device for leakage electric field in the process of foundation pit pumping test

**DOI:** 10.1371/journal.pone.0328727

**Published:** 2025-08-20

**Authors:** Yufeng Chen, Hui Chen, Jiayong Yan, Yuexin You, Suiming Liu

**Affiliations:** 1 School of Geophysics and Measurement-control Technology, East China University of Technology, Nanchang, China; 2 Chinese Academy of Geological Sciences, Beijing, China; 3 Engineering Research Center for Seismic Disaster Prevention and Engineering Geological Disaster Detection of Jiangxi Province, Nanchang, China; China University of Mining and Technology, CHINA

## Abstract

Early detection of leakage in foundation pit retaining structures during excavation is critical for ensuring both construction safety and the integrity of adjacent buildings. Conventional surface direct current methods suffer from poor resolution, low interference to resistance, and limited capability in pinpointing leakage locations. To achieve accurate leakage identification and enhance the quality control of major engineering projects, this study first establishes a coupled electrokinetic-steady electric field response mechanism by integrating the naturally occurring electric field from electrokinetic effects in leakage zones with artificial steady electric fields during pumping tests. Fundamental governing equations were derived to characterize the leakage-induced electric field. Subsequently, a novel leakage detection system combining pumping tests with electric field measurements was developed. This system employs current injection through groundwater observation wells while monitoring surface potential distribution patterns during dewatering processes, enabling rapid localization of leakage points. A 3D finite element-infinite element coupled numerical model was established to perform forward modeling, systematically investigating the influences of leakage channel spatial distribution, and system parameters on electric field responses. Laboratory-scale physical model tests demonstrated that the proposed method significantly amplifies potential anomalies at leakage locations through pumping-induced groundwater flow. Results indicate that the integrated pumping-electric field detection system effectively reveals leakage-induced potential anomalies. The electrokinetic effect generated by groundwater movement through leakage channels during pumping operations substantially enhances detection signals, thereby improving both the accuracy and efficiency of leakage identification. This advancement provides a robust technical solution for quality assurance in deep excavation engineering.

## 1 Introduction

The intensive development of urban underground spaces in recent years has driven deep foundation pit engineering toward unprecedented scales and depths. Projects such as underground railways, transportation hubs, and ultra-high-rise buildings frequently involve complex leakage risks in foundation pit enclosures, posing significant challenges to structural safety [1,2]. Statistical analyses indicate that over 60% of foundation pit accidents stem from soil loss and structural instability triggered by seepage [3,4]. The concealed nature and dynamic evolution of seepage pathways further complicate detection, rendering traditional methods inadequate for timely risk mitigation [5].

Current leakage detection methodologies fall into two categories: geophysical techniques and hydrogeological analyses. Geophysical approaches rely on identifying physical anomalies in leakage zones, employing methods such as temperature tracer monitoring [6,7], the electrochemical response method (ECR) [8], flow field analysis [9,10], electromagnetic methods [11,12], and electrical resistivity tomography [13,14]. Notably, distributed fiber optic sensing (DFOS) has emerged as a promising tool for real-time strain field monitoring; however, its spatial resolution remains constrained by fiber deployment density and directional sensitivity [15]. Hydrogeological methods, such as pumping tests, evaluate the integrity of hydraulic barriers [16], yet conventional observation wells often fail to capture intricate seepage paths [16–18]. While resistivity-based techniques dominate due to their sensitivity to low-resistivity anomalies, their efficacy is limited by shallow signal-to-noise ratios and three-dimensional electric field distortions, particularly in scenarios with high depth-to-diameter ratios [19].

To address these limitations, recent studies have explored multi-physics coupling mechanisms to enhance detection accuracy. For instance, CFD-DEM simulations elucidate the interplay between seepage fields and particle migration during leakage [20], while cohesive zone modeling reveals the dynamic evolution of seepage-induced joint fractures [21]. These advancements highlight the untapped potential of electrokinetic effects—electrical signals generated by fluid movement during pumping [22]—as indicators of seepage characteristics. However, existing research predominantly focuses on static leakage fields, neglecting systematic investigations into the dynamic electric responses under pumping perturbations. Moreover, guidelines for optimizing coupled well-surface observation systems remain underdeveloped.

This study introduces a paradigm shift from static detection to synergistic pumping excitation–electrokinetic response analysis. First, a three-dimensional unstructured finite-element–infinite-element coupled model is developed to simulate the spatiotemporal evolution of electric fields within leakage channels during pumping, grounded in leakage-electric field coupling theory. Second, a novel observation system is engineered to extract high-fidelity leakage-induced electric signals from both subsurface and surface environments. Finally, numerical simulations and physical experiments are integrated to establish quantitative relationships between leakage location/scale and electric field responses, providing robust inversion constraints for practical applications. By advancing the theoretical framework of kinetic effects in engineering detection, this work offers an innovative solution for precise leakage localization in complex geotechnical environments.

## 2 Foundation pit leakage electric field response mechanism

### 2.1 Current aggregation effect

**Fig 1:**
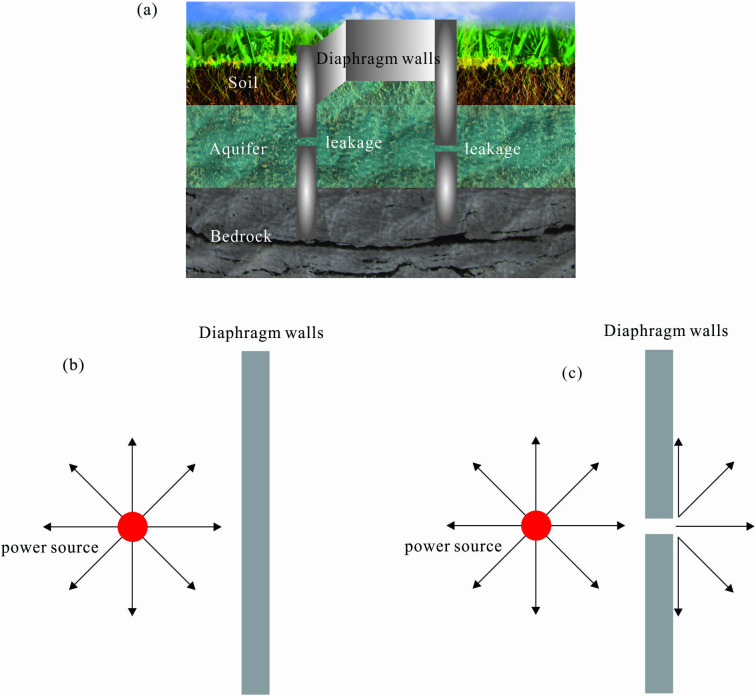
Schematic diagrams illustrating the leakage of the pit enclosure structure and the current field distribution due to the current aggregation effect. (a) Schematic diagram of the leakage of the pit enclosure structure, (b) electric field line distribution in the absence of leakage, (c) electric field line distribution in the presence of leakage.


Structural defects in pit enclosures facilitate the formation of enrichment conductive channels (Fig 1a). These channels exhibit significantly reduced resistivity compared to the surrounding medium. When subjected to a homogeneous electric field, the low-resistivity zones induce a Current Aggregation Effect (CCE) as shown in Fig 1c. This phenomenon manifests as localized potential elevation in moisture-containing regions, resulting in measurable surface potential anomalies. Conversely, intact pit enclosures without leakage pathways (Fig 1b) demonstrate uniform current distribution through the structural matrix, maintaining normal potential distribution without detectable anomalies. The DC excitation system applied through subsurface electrodes establishes a quasi-stationary current field governed by Ohm’s law:

𝐉=−σ∇φ (1)

where J denotes the current density vector, *φ* represents the electric potential, and *σ* signifies the conductivity of the subsurface medium. In accordance with the principle of charge conservation, the current continuity equation can be expressed as:


∇·𝐉=0 (2)

Substituting Equation (1) into Equation (2) results in the Laplace-transformed differential equation that governs the stabilized current field, which can be expressed as:


∇(σ∇φ)=Iδ(r) (3)

where I represents the current intensity of the point source, δ denotes the Dirac delta function, and r signifies the distance from the observation point to the point source. Equation (3) is solved numerically subject to the following boundary conditions:


$$\varphi =0\begin{array}{cc} {}*20c & on\begin{array}{cc} {}*20c & \;\Gamma = \end{array} \end{array}\Gamma_{D}$$ (4)

$$-𝐧\cdot 𝐉=0\begin{array}{cc} {}*20c & on\begin{array}{cc} {}*20c & \;\Gamma = \end{array} \end{array}\Gamma_{N}$$ (5)

Γ_*D*_ and Γ_*N*_ represent the Dirichlet and Neumann boundaries, respectively, while **n** denotes the outward-pointing unit normal vector to Γ_*N*_

### 2.2 Electrokinetic effects

**Fig 2:**
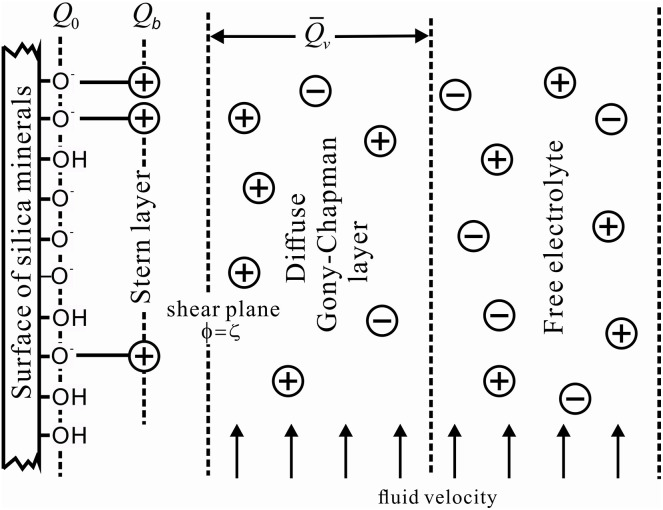
Illustration of double electrolayer in porous dielectrics (after Jougnot et al., 2015 [28]).

When leakage occurs in the underground enclosure of a foundation pit, groundwater infiltrates the pit through the leakage channel during the dewatering process. This infiltration induces a self-potential, which is categorized as an electrokinetic phenomenon [23]. In subsurface porous media, electric double layers form within the pore fluids [24]. These layers consist of a compact layer and a diffuse layer. The compact layer comprises ions adsorbed onto the mineral interface to neutralize surface charges, while the diffuse layer contains mobile excess charges (Fig 2). As fluid flow mobilizes the charges within the diffuse layer, a kinetic electric current is generated [25–27], leading to the generation of a self-potential (SP) signal.

The classical formulation of electrokinetic processes in subsurface porous media is based on a linear coupling of two constitutive equations for current density and seepage velocity [29], given by


$$𝐉=-\sigma \nabla \varphi -L\nabla \left(p-\rho_{f}g\right)$$ (6)

$$𝐮=-L\nabla \varphi -\frac{k}{\eta_{f}}\nabla \left(p-\rho_{f}g\right)$$ (7)

$$C={\left(\frac{\partial \varphi }{\partial p}\right)}_{j=0}=-\frac{L}{\sigma }\approx \frac{\varepsilon_{f}\zeta }{\eta_{f}\sigma_{f}}$$ (8)

where **u** represents the Darcy velocity (m/s), *p* denotes the pore fluid pressure (Pa), g is the gravitational acceleration vector (m/s^2^), *ρ*_*f*_ signifies the mass density of the pore water (kg/m^-3^), *η*_*f*_ indicates the dynamic viscosity (P·s), L represents the coupling coefficient of the flow current (m^2^/V·s), *k* stands for the permeability (m^2^), *C* is the coupling coefficient of the flow potential (V/Pa), *ε*_*f*_ is the dielectric constant of the pore water, and *ζ* denotes the zeta potential (V). By neglecting the electro-osmotic contribution in the Darcy equation [30], Equation (6) can be reformulated to directly account for the Darcy velocity **u**, as follows [31]:


$$𝐉=-\sigma \nabla \varphi +{\bar{Q}}_{v}𝐮$$ (9)

The charge density per unit pore volume in the pore water (in C/m^3^) is located within the electrodiffusive layer, and this quantity is correlated with the permeability coefficient [30,32].


$$\log_{10}{\bar{Q}}_{v}=-3.49-0.82\log_{10}K$$ (10)

Equation (7) can be simplified:


$$𝐮=-\frac{k}{\eta_{f}}\nabla \left(p-\rho_{f}g\right)=-K\nabla (H+z)$$ (11)

where *K* is the hydraulic conductivity (m/s), *H* is the pressure head (m), and *z* is the elevation (m). The solution of the differential equation for the self-potential *φ* is obtained by substituting equation (9) into equation (2):


$$\nabla \cdot (\sigma \nabla \varphi )=\nabla \cdot \left({\bar{Q}}_{v}𝐮\right)$$ (12)

Equation (12) is numerically solved using Dirichlet and Neumann boundary conditions. When an artificial DC electric field is applied to the inner and outer boundaries of the foundation pit, and the flow current density generated by groundwater movement is accounted for, the total current density can be expressed based on the principle of electric field superposition as:


$$𝐉={𝐉}_{ar}+{𝐉}_{sp}$$ (13)

where J_*ar*_ is the current density of the artificial current field, the flow current density ${𝐉}_{sp}={\bar{Q}}_{v}u$, and the solution of the differential equation for the total potential *φ* after the superposition of the artificial DC field and the self-electric field is obtained by substituting equation (13) into equation (2):


$$\nabla \cdot (\sigma \nabla \varphi )=\nabla \cdot \left({𝐉}_{ar}+{𝐉}_{sp}\right)$$ (14)

Equation (14) can still be solved numerically using the Delicacy and Neumann boundaries.

## 3 3D numerical simulation and results

### 3.1 Finite element 3D simulation based on unstructured meshes

To examine the three-dimensional response characteristics of the foundation pit leakage electric field, numerical simulations of the artificial DC electric field, seepage field, and electrokinetic process were performed utilizing the **unstructured** mesh finite element method.

**Fig 3:**
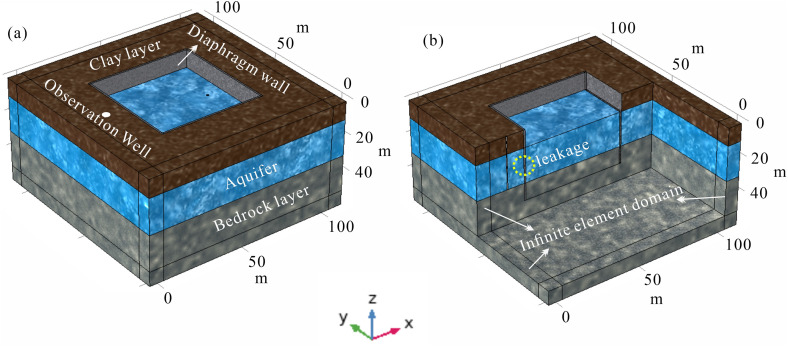
Geometry of a typical foundation pit leakage model.

Initially, a geometric model of a typical foundation pit leakage scenario was established (Fig 3). The background field was defined with dimensions of 116m × 116m × 58m. The foundation pit was modeled as a rectangular prism measuring 60m in length and width, with a burial depth of 40m. The stratigraphic structure, representing real-world geological complexity, was divided into three distinct layers: an upper relatively dry clay layer, an intermediate water-bearing layer where leakage typically occurs, and a bottom water-isolating bedrock layer. Consequently, the pit model was constructed as a three-layer medium with the following properties: an upper clay layer 10m thick, a middle aquifer 20m thick, and a bottom bedrock layer 10m thick. The thickness of the foundation pit enclosure is between 0.5 and 1.2m, so we set the thickness of the foundation pit enclosure to 1m. A cylindrical leakage channel with a radius of 0.25m was utilized to simulate leakage pathways. Additionally, hydrological observation wells, typically situated outside the foundation pit, were represented as cylinders with a radius of 0.25m and a height of 30m. To simulate the infinite background field, an 8 cm-thick infinite element domain was applied around the model boundary. This configuration ensures accurate simulation of electric and flow field interactions in the presence of leakage within the foundation pit.

**Fig 4:**
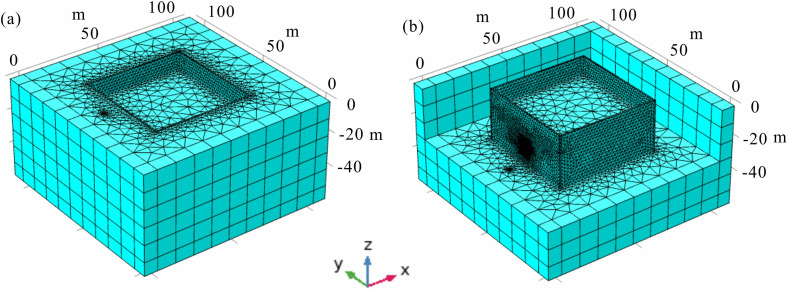
Discretized schematic of a typical deep foundation pit leakage model.

After constructing the geometric model of the deep foundation pit, it is imperative to discretize the various solution domains. Initially, the infinite element domain is discretized using swept meshing. The primary objective of this domain is to approximate an unbounded region for solving diffusion-type **governing** equations, such as those governing the electric field. Swept meshing is employed here to effectively simulate the infinite extent. For the remaining regions, tetrahedral meshing is utilized due to its high adaptability, superior automation during mesh generation, and suitability for handling complex and irregular geometries. Considering the relatively small scale of the leakage channel and observation well compared to the overall background model, localized mesh refinement is applied to these features to ensure accuracy. A detailed representation of the mesh discretization for the foundation pit leakage model is provided in Fig 4.

**Fig 5:**
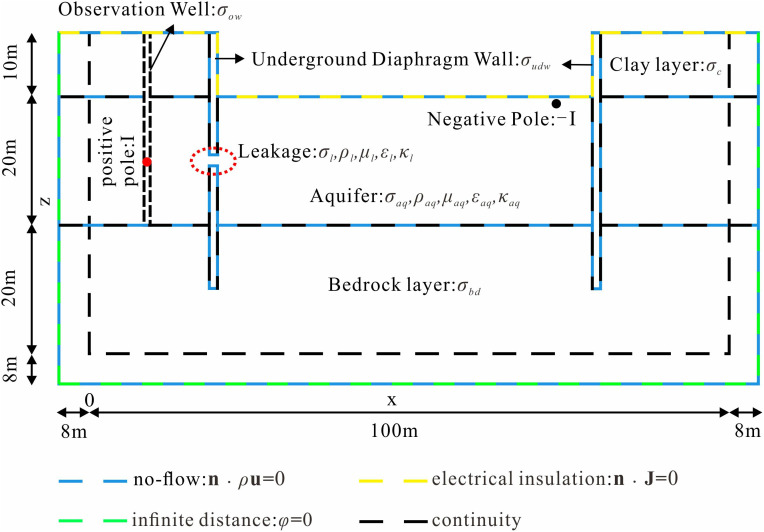
Diagram of physical attributes and boundary condition settings of the foundation pit leakage model.

Upon completion of the discretization of the foundation pit leakage model, the corresponding physical properties and boundary conditions were assigned, followed by the finite element solution process. Fig 5 provides an overview of the physical properties and boundary condition configurations applied to the model. For the seepage field, it is assumed that fluid flow occurs exclusively within the aquifer. Therefore, no-flow boundary conditions are imposed at the interfaces between the aquifer and the clay layer and between the aquifer and the bedrock layer. Within the pit enclosure, flow is confined to the seepage channel, while all other boundaries are designated as no-flow. A hydraulic head difference is then applied between the inner and outer sides of the pit to compute the flow field distribution. To solve for the steady-state current field, insulating boundary conditions are applied at the interface between the model and the air, while far-field boundary conditions are assigned to the remaining external boundaries. Continuity boundary conditions are applied to the internal boundaries. By introducing a current source on the positive and negative sides of the model, an artificially stimulated DC electric field is generated. The self-potential generated by the electrokinetic effect is determined using the seepage velocity from the flow field solution, which is coupled into the current density term to compute the model’s self-electric field. Table 1 summarizes the physical property parameters configured for the pit leakage model.

**Table 1:** Parameters of foundation pit leakage model.

Solution domain	σ (S/m)	μ (Pa·s)	ε	*K* (m/s)
**Clay layer**	1 × 10^−2^	\	\	\
**Aquifer**	2 × 10^−2^	1.3 × 10^−3^	3.5 × 10^−1^	2.0 × 10^−2^
**Bedrock**	5 × 10^−4^	\	\	\
**Leak path**	1 × 10^−1^	1.3 × 10^−3^	8.5 × 10^−1^	4.0 × 10^−1^
**Observation well**	2 × 10^−2^	1.3 × 10^−3^	\	\
**foundation pit enclosure structure**	5 × 10^−3^	\	\	\

### 3.2 Simulation results of the seepage field

**Fig 6:**
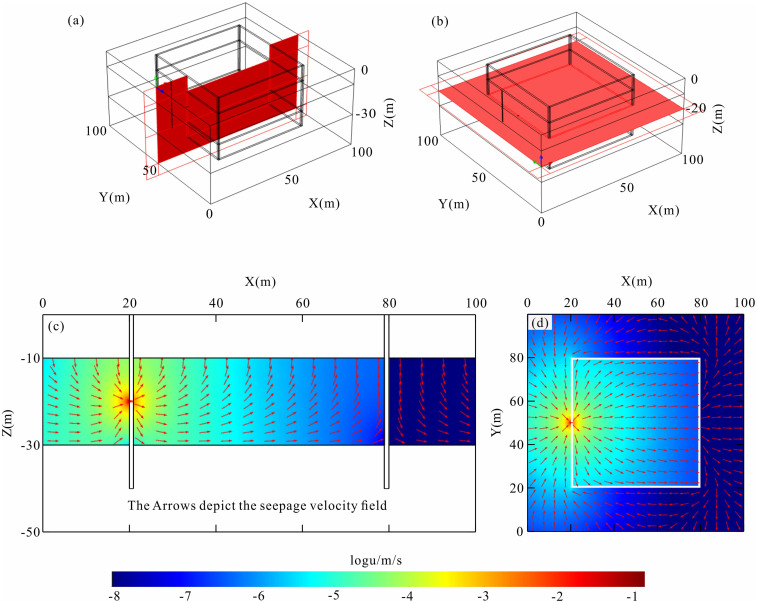
Simulation results of seepage field of foundation pit seepage model. (a) Section schematic of foundation pit leakage model; (b) Plan schematic of foundation pit leakage model; (c) Seepage velocity section of foundation pit leakage model; (d) Seepage velocity plan of foundation pit leakage model.

Fig 6 illustrates the simulated results of the seepage field within the foundation pit. In this visualization, red denotes areas with high seepage rates, blue indicates regions of Low seepage velocity, and arrows represent the vectors of the flow field. During the dewatering process, groundwater flows from outside the foundation pit into its interior through seepage channels, driven by the pressure gradient. When the pressure difference between the interior and exterior of the pit is zero, the water level inside the pit will be in equilibrium with the external groundwater level. To induce the flow of groundwater into the foundation pit via the leakage holes, it is necessary to pump water from the dewatering wells installed within the foundation pit. This action creates a hydraulic head difference between the inside and outside of the foundation pit, thereby driving the flow of groundwater towards the foundation pit. As depicted in Fig 6, groundwater converges towards the interior of the foundation pit from all directions due to the hydraulic head difference. The highest flow velocities are observed in the vicinity of the seepage channels, with an average velocity of 0.014 m/s recorded in these areas. No flow field exists in the clay and impermeable layers, as these zones are designated as impermeable in the model.

### 3.3 Simulation results of leakage electric field

**Fig 7:**
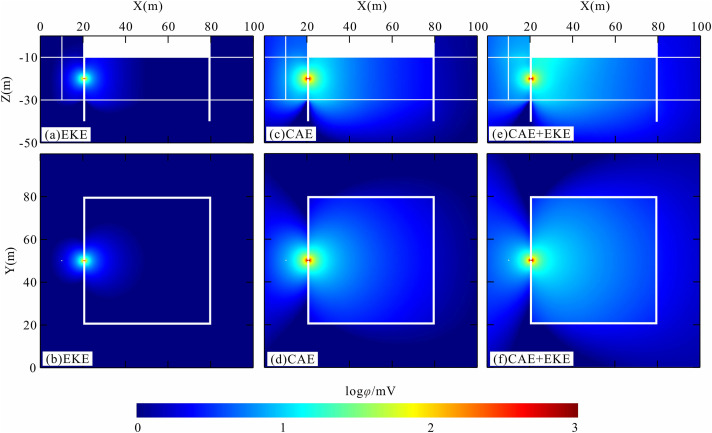
3D simulation results of anomalous potential for pit leakage modeling.

Fig 7 presents the three-dimensional numerical simulation results of the anomalous potential within the foundation pit leakage model. The anomalous potential is quantified as the deviation derived from subtracting the potential values of the no-leakage scenario from those of the leakage scenario. In the visualization, red signifies regions with high anomalous potential, whereas blue denotes areas of low or negligible anomaly. Fig 7(a, b) illustrate the anomalous potentials generated by the electrokinetic effect during pit leakage events. Under stationary groundwater conditions, the system maintains electrical equilibrium, resulting in the absence of an external electric field. However, when leakage occurs through defects in the pit enclosure, pressurized groundwater flows into the pit via these leakage points. This flow mobilizes electric charges within the diffusion layer, inducing an electrokinetic effect that generates flow currents and self-potential (SP) signals, thereby producing anomalous potentials along the leakage pathways. Fig 7(c, d) depict the anomalous potentials resulting from the current aggregation effect in the presence of leakage. When an electric dipole field is established between the observation well and the pit, current emanates from the positive pole and returns to the negative pole through the pit enclosure. Should the pit enclosure contain defects that form water-rich channels with low resistivity, the current aggregation effect within the uniform electric field amplifies the potential in these leakage areas. The resultant anomalous potential exhibits a butterfly-shaped distribution centered on the leakage channel, gradually attenuating outward. Fig 7(e, f) illustrate the anomalous potentials when both the current aggregation effect and electrokinetic effect are taken into account. It is apparent that the anomalous potential regions in Fig 7(e, f) are substantially larger than those in Fig 7(a, d). This enlargement is attributed to the superposition of electric fields generated by the collector and electrokinetic effects, thereby enhancing the overall leakage-induced electric field.

**Fig 8:**
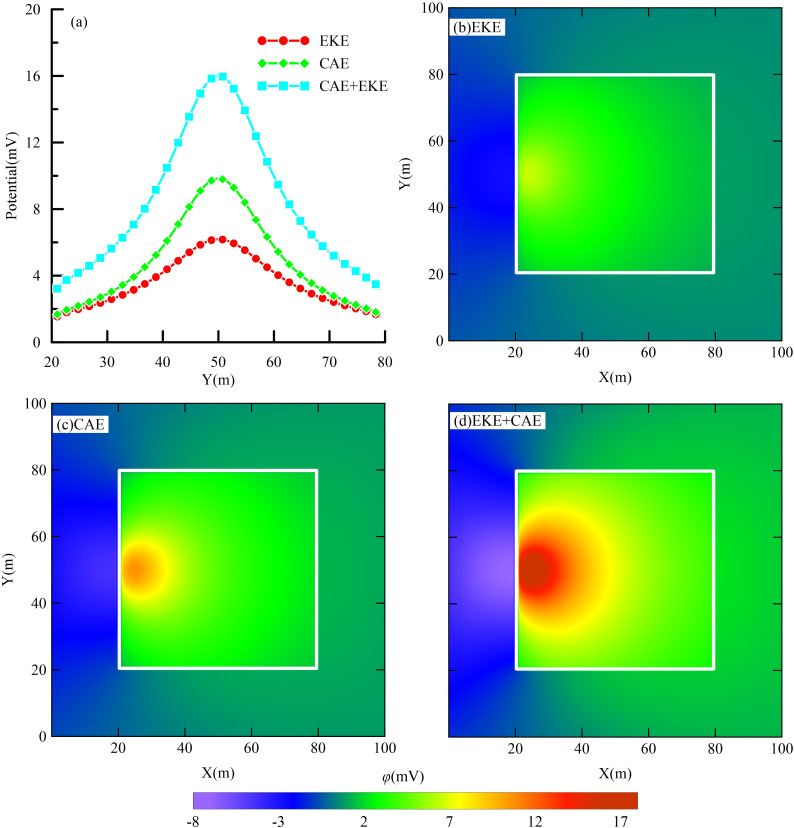
Measured line anomaly curves and surface anomaly potential planes for pit leakage modeling.

Fig 8 illustrates the anomaly curve along the measurement line and the surface anomalous potential distribution for the foundation pit leakage model. The measurement line is situated 2 meters to the right of and parallel to the pit enclosure wall (i.e., X = 23 m). The electric field generated by the foundation pit leakage induces an anomalous potential within the leakage channel, which manifests as a corresponding image on the pit surface. The location of this surface anomalous potential varies depending on the relative position between the signal source and the actual leakage channel. Therefore, monitoring surface potential anomalies facilitates the accurate identification of potential leakage locations.

Fig 8b illustrates the surface potential anomalies induced by the leakage electric field due to the electrokinetic effect. During seepage, groundwater infiltrates the pit via leakage points, carrying positive ions from the solution. This transport results in an accumulation of negative charges outside the pit (upstream of the water flow), leading to a negative potential, while inside the pit, positive ions accumulate, creating a positive potential. The observed self-potential (SP) signal attributed to the electrokinetic effect on the surface measures approximately 7 mV, with an average seepage velocity of 0.014 m/s within the leakage channel.

Fig 8c illustrates the surface potential anomalies induced by the current aggregation effect. Notably, positive potential anomalies are observed within the foundation pit, whereas negative anomalies are evident outside, with anomaly values approximating 10 mV. In Fig 8d, the synergistic impact of both the collector effect and the electrokinetic effect on the leakage electric field results in surface potential anomalies. These anomalies are significantly more pronounced, with potential values reaching approximately 17 mV. This amplification is attributed to the superposition of electric fields generated by the collector and electrokinetic effects.

The anomaly curves along the measurement line (Fig 8a) visually illustrate the contributions of both the collector effect and the electrokinetic effect to the foundation pit leakage electric field. It is clear that the current aggregation effect significantly influences the formation of the leakage electric field. Nevertheless, the self-potential (SP) signals generated by the electrokinetic effect remain significant. The combined influence of the collector and electrokinetic effects enhances the potential anomaly signal, thereby improving the detection and diagnosis of concealed foundation pit leakage issues.

## 4 Response characteristics of foundation pit leakage electric field detection system

### 4.1 Foundation pit leakage electric field detection device

**Fig 9:**
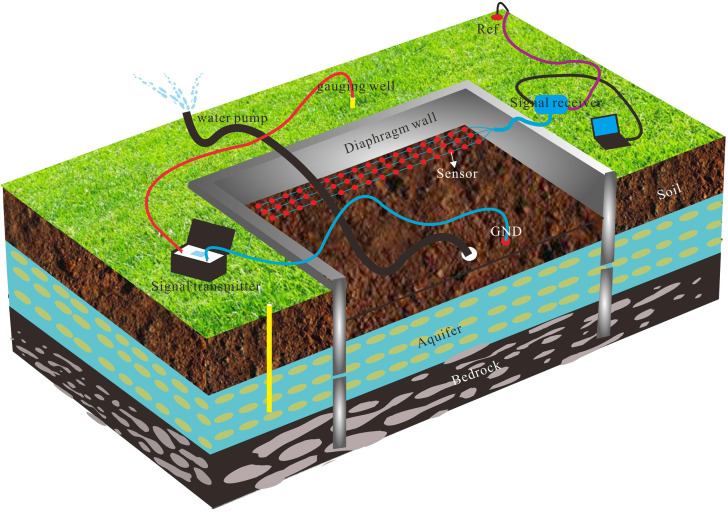
Diagram of a foundation pit leakage electric field detection device combined with pumping test.

To address the challenges of low accuracy and weak signal strength inherent in traditional ground-based DC electrical exploration methods, this study introduces a novel foundation pit leakage electric field detection device integrated with a pumping tests. This device is designed based on the formation mechanism and three-dimensional response characteristics of the leakage electric field (as illustrated in Fig 9). By utilizing gauging well outside the foundation pit, the device establishes an artificial direct current electric field to ensure that the current flows from the exterior of the pit toward the interior. Within the pit, strategically arranged non-polarized electrodes measure potential or potential gradients, enabling precise localization of leakage pathways. In the absence of leakage within the enclosing wall, the wall exhibits uniform conductivity, allowing the current to pass through it evenly, resulting in no detectable potential anomalies within the measurement electrode distribution area. However, when leakage occurs, the current converges and flows through the wall at the leakage points, creating distinct potential anomalies observable within the measurement area. Additionally, self-potential signals arise from the electrokinetic effect induced by groundwater flow. Simultaneously monitoring the leakage electric field signal during the precipitation phase of the pumping test amplifies the potential anomaly signal generated by fluid movement in the leakage channels. This enhancement significantly improves the sensitivity and accuracy of detecting leakage pathways.

### 4.2 Key parameters of the detection device

**Fig 10:**
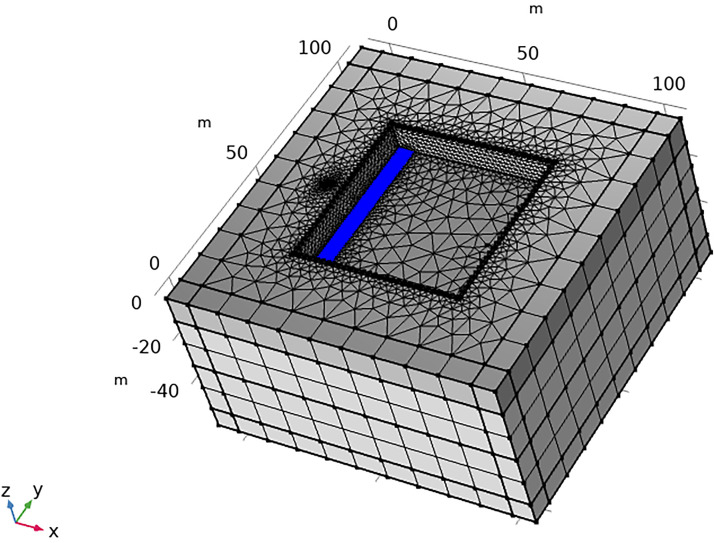
Discretized schematic of the foundation pit leakage electric field model.

To further refine the parameters of the foundation pit leakage electric field detection device, this study employs three-dimensional numerical simulations to investigate critical parameters such as the magnitude of the emitting current, the burial depth of the emitting point, the distance between the emitting positive electrode and the foundation pit maintenance structure, and the seepage velocity. By analyzing the characteristics of the electric field response, key parameters for optimizing the detection device are identified. Additionally, the influence of factors including the size, burial depth, horizontal position, and leakage rate of the seepage channel is evaluated to provide theoretical guidance for the localization and interpretation of leakage potentials. The model dimensions align with those of the typical foundation pit leakage electric field model illustrated in Fig 3, while all other parameters remain constant except for variations in the detection device parameters under investigation. Previous studies have shown that leakage through the pit’s cutoff curtain generates positive potential anomalies on the inner side of the pit. Consequently, the area adjacent to the cutoff curtain is designated as the observation region (highlighted in blue in Fig 10), with a refined grid in this area to enhance simulation accuracy.

Effects of emission current

#### 4.2.1 Effect of emission current.

**Fig 11:**
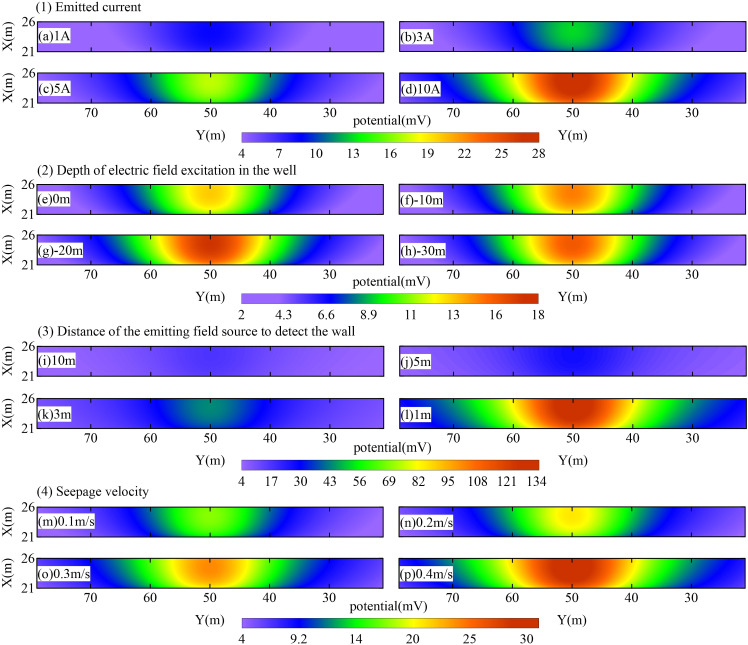
Results of anomalous potential response of the observation area with different detection device parameters.

To examine the impact of emission current on the detection device, this study systematically evaluates the electric field response under different emission current conditions. The emission current is set to 1 A, 3 A, 5 A, and 10 A, respectively, while all other parameters are held constant. The specific configurations are as follows: the positive emission position is located at (15 m, 50, −20), the negative emission position at (70 m, 50, −10), and the leakage channel position at (20 m, 50, −20). Fig 11(1) illustrates the anomalous potential response within the observation region for various emission current settings. The results demonstrate that the anomalous potential in the observation region increases substantially with higher emission currents. Therefore, during actual leakage detection of foundation pit enclosures, when the anomalous signal is weak or field interference is significant, increasing the transmission current can enhance the potential signal. This method effectively mitigates interference and improves detection accuracy.

#### 4.2.2 Effect of emitting positive electrode burial depth.

To examine the impact of the burial depth of the transmitting positive electrode on the electric field response and to determine the optimal burial depth, a series of models with varying electrode depths were analyzed. The burial depth of the positive electrode was systematically adjusted to different positions in the model—specifically, at coordinates (15 m, 50, 0), (15 m, 50, −10), (15 m, 50, −20), and (15 m, 50, −30)—while all other parameters remained constant. The transmitting negative electrode was fixed at (70 m, 50, −10), the leakage channel was positioned at (20 m, 50, −20), and the transmitter current magnitude was set to 5 A. Based on these parameters, the electric field response under varying positive electrode burial depths was computed. Fig 11(2) illustrates the anomalous potential response in the observation region for different burial depths of the transmitting positive electrode. The results indicate that the anomalous potential profiles across the observation region exhibit a consistent pattern of variation irrespective of the electrode depth, with significant potential anomalies observed above the seepage channel in all cases. However, the influence of the positive electrode burial depth on the anomalous potential response in the observation area is minimal. Notably, the electric field response within the aquifer is more pronounced compared to that in the clay layer. Therefore, for practical observation purposes, it is recommended to stimulate the electric field within the aquifer. Additionally, the drilling depth of observation wells should only extend to the aquifer to efficiently meet observation requirements.

#### 4.2.3 Effect of the distance of the transmitter positive pole from the foundation pit enclosure structure.

In this analytical model, the distance between the observation wells and the pit enclosure structure is systematically varied to evaluate its impact on the electric field response. The positions of the transmitter’s positive electrodes are set at (10 m, 50 m, −20 m), (15 m, 50 m, −20 m), (17 m, 50 m, −20 m), and (19 m, 50 m, −20 m), corresponding to distances of 10 m, 5 m, 3 m, and 1 m from the pit enclosure structure, respectively. All other parameters remain constant: the negative electrode is positioned at (70 m, 50 m, −10 m), the leakage channel is located at (20 m, 50 m, −20 m), and the current emission strength is maintained at 5 A. Numerical simulations were conducted to assess the electric field response for varying distances between the transmitter’s positive electrode and the foundation pit enclosure structure. The results, as illustrated in Fig 11(3), indicate a significant decrease in anomalous potential within the observation area as the transmitter’s positive electrode moves farther from the pit enclosure structure. This finding underscores the importance of positioning the transmitter as close as possible to the pit enclosure structure in practical applications to enhance observation accuracy.

#### 4.2.4 Effect of seepage velocity.

The seepage velocity exhibits a direct proportional relationship with the self-potential, which is a critical parameter for leak detection. This study investigates this relationship by systematically varying the seepage velocity in the model (0.1 m/s, 0.2 m/s, 0.3 m/s, and 0.4 m/s) while holding other parameters constant. In this experimental setup, the positive transmitter is located at coordinates (15 m, 50 m, −20 m), the negative transmitter at (70 m, 50 m, −10 m), and the leakage channel at (20 m, 50 m, −20 m). The electric field response is evaluated for different transmitter currents. Fig 11(4) illustrates the anomalous potential response within the observation region for various seepage velocity models. The results indicate that the anomalous potential increases as the seepage velocity rises, suggesting that seepage velocity significantly influences the observation region. In practical applications of pit envelope leakage detection, integrating the process with a pumping test and increasing the precipitation rate can enhance seepage velocity. This approach can further amplify the anomalous potential signal, thereby improving the effectiveness of the detection process.

### 4.3 Response regularity analysis of leakage pathway

#### 4.3.1 Effect of burial depth of the leakage pathway.

**Fig 12:**
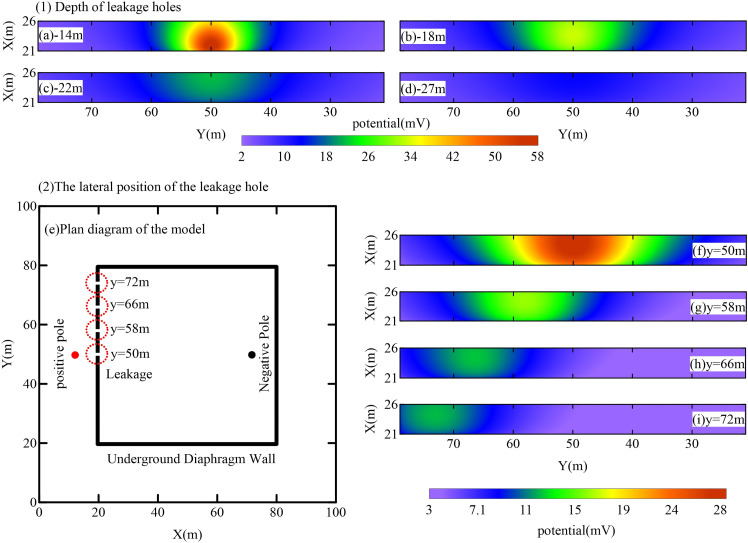
Results of anomalous potential response in the observation area for different seepage depths and horizontal locations.

To assess the capability of the foundation pit leak detector in identifying leakage pathways at varying depths, models with different leakage pathway depths were analyzed. The specific locations of the leakage pathways in the models are as follows: (20 m, 50 m, −14 m), (20 m, 50 m, −18 m), (20 m, 50 m, −22 m), and (20 m, 50 m, −27 m). All other parameters remained constant. The positive transmitter was positioned at (15 m, 50 m, −20 m), while the negative transmitter was located at (70 m, 50 m, −10 m). The transmitter current was set to 5 A. The electric field response for each leakage pathway at varying depths was computed. Fig 12(1) illustrates the anomalous potential response within the observation area for the different burial depth models. Analysis indicates that as the depth of the leakage pathway increases, the anomalous potential signal within the observation area diminishes progressively and significantly. Shallow leakage pathways generate higher anomalous potential values with a more concentrated anomaly area. However, when the depth of the leakage pathway exceeds 22 m, the anomalous potential signal becomes too weak for reliable observation.

#### 4.3.2 Effect of horizontal position of leakage paths.

The intensity of the current field decreases with increasing distance from the source, indicating that the detection capability of a single emitted field source for foundation pit enclosures is constrained in range. To evaluate the horizontal control range of the leakage pathway, the horizontal positions of the leakage path were varied (as shown in Fig 12(2e)) and set at coordinates (20 m, 50 m, −20 m), (20 m, 58 m, −20 m), (20 m, 66 m, −20 m), and (20 m, 72 m, −20 m). With all other model parameters held constant, the positions of the positive and negative emitters were fixed at (15 m, 50 m, −20 m) and (70 m, 50 m, −10 m), respectively, while the emitter current was maintained at 5 A. The electric field responses for different leakage path positions were computed, and the results are presented in Fig 12(2). Analysis reveals that the anomalous potential response within the observation region diminishes progressively as the leakage pathway moves farther from the central position of the field source. When the leakage path exceeds a distance of 20 m from the field source center, the anomalous potential signal becomes too weak to be effectively detected. Therefore, in practical detection scenarios, the effective detection range of a single emitting field source should be limited to within 40 m of the pit enclosure. For larger pit enclosures, multiple artificial field sources can be employed to ensure comprehensive coverage and mitigate the issue of limited detection range.

#### 4.3.3 Effect of the number of leakage paths.

**Fig 13:**
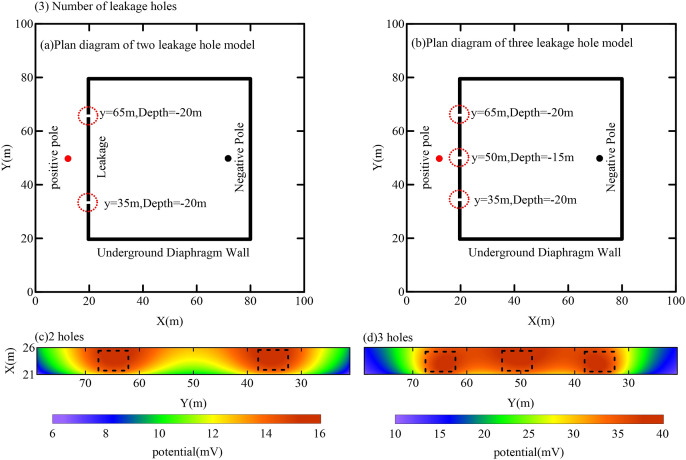
Results of anomalous potential response in the observation area for different numbers of leak paths.

To assess the detection capability of the foundation pit leak detector for varying numbers of leakage paths, an analysis was conducted by systematically altering the number of leakage paths while holding all other parameters constant. Each leakage path had a radius of 0.25 m. The transmitter electrode positions were set at (15 m, 50 m, −20 m) for the positive electrode and (70 m, 50 m, −10 m) for the negative electrode, with a constant current of 5 A. For the two-path scenario, the leakage positions were defined as (20 m, 65 m, −20 m) and (20 m, 35 m, −20 m). In the three-path scenario, the positions were (20 m, 65 m, −20 m), (20 m, 50 m, −15 m), and (20 m, 35 m, −20 m). Based on these configurations, the electric field responses were calculated for each scenario. The results, illustrated in Fig 13, depict the anomalous potential responses in the observation area under different numbers of leakage paths. When two leakage paths are present, two distinct potential anomaly regions are observed. In the case of three leakage paths, three anomaly regions are detected; however, these regions appear spatially contiguous, leading to a reduced ability to clearly distinguish areas of high potential anomalies. These findings indicate that the method is effective in identifying multiple leakage paths within the pit enclosure structure. Nevertheless, further research and optimization are necessary to address complex scenarios involving densely distributed leakage pathways.

## 5 Laboratory experiments

### 5.1 Materials and methods

**Fig 14:**
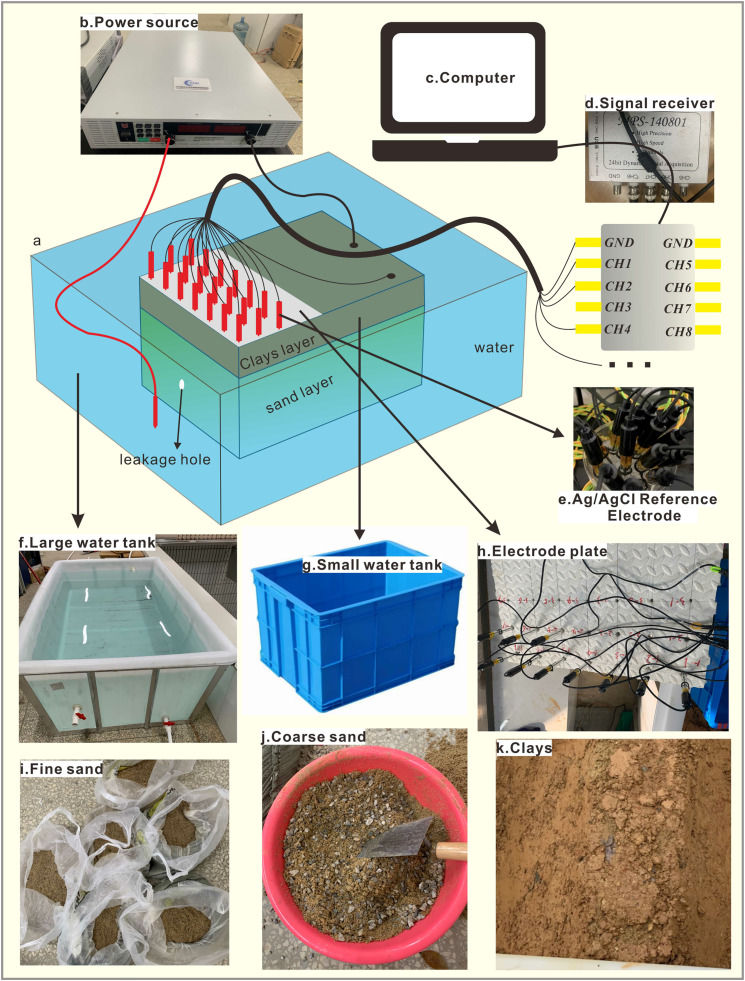
Schematic diagram of the experimental setup and materials.

To verify the effectiveness of the pit leakage electric field observation device, this study constructed a three-dimensional physical modeling test system (as shown in Fig 14). The experimental system comprises an excitation-measurement unit and a pit hydrogeological simulation unit, which are configured as follows: (1) Electromethod observation system: the excitation unit employs a self-developed high-precision programmable DC power supply (Fig 14b) to establish an artificial electric field via a copper transmitter electrode. The measurement unit is equipped with a 24-bit high-resolution potential signal acquisition system (Fig 14d) and utilizes Ag/AgCl reference electrodes (Fig 14e) for potential measurements. Electrodes were pre-treated according to specifications. Prior to the experiment, the protective cover was removed, and air bubbles in the electrolyte cavity were eliminated by vertical shaking after immersion in saturated NaCl solution for 2 hours. (2) Hydrogeological simulation system with a double-box structure designed to simulate the pit-peripheral aquifer system (as depicted in Fig 14f and g). The primary tank (200 × 115 × 60 cm) was filled with tap water to simulate a natural aquifer. A hydraulic connection was established between the main tank and the built-in pit model tank (56 × 85 × 45 cm) via a controlled leakage hole with a diameter of 1.4 cm. The model box was designed with stratified filling, where the bottom permeable layer consisted of 30 cm thick fine sand (as shown in Fig 14i). The center of the leakage channel was replaced by a column of coarse sand (Fig 14j), while the top impermeable layer was filled with 15 cm thick compacted clay (Fig 14k). Prior to the experiment, a leakage field was established using a water level adjustment device, maintaining a head difference of 40 cm between the tanks. Leakage holes were sealed with waterproof sealant to ensure no leakage in the initial state. In the testing procedure, background field measurements were conducted using a fixed-source power supply method (constant voltage: 10V), followed by the opening of the leakage holes and dynamic potential monitoring. All experiments were performed in an electromagnetic shielding room, and data were synchronously collected and stored via the LabVIEW platform.

### 5.2 Results

**Fig 15:**
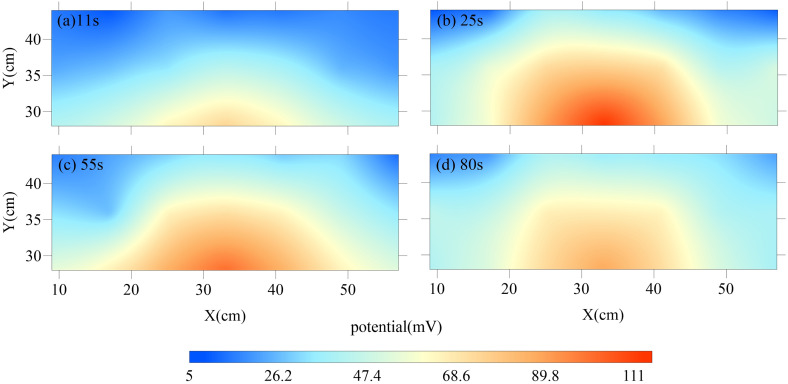
Results of laboratory physical modeling tests.

Dynamic potential monitoring was conducted during leakage hole activation under steady-state background field conditions (DC supply voltage: 10 V, sampling rate: 10 Hz, duration: 180 s). Following data preprocessing, the spatiotemporal evolution of the normalized potential anomaly field (ΔV = V(t) - V_min_) was derived, as illustrated in Fig 15. The color-coded intensity (0–113 mV) reflects the magnitude of the potential anomaly, with red and blue denoting high and low potentials, respectively.

Experimental observations identified three distinct stages of Leakage-electric field coupling (Fig 15a–c):

(1) Leakage Initiation Stage (t = 0–11 s):

At t = 11 s (Fig 15a), the abrupt opening of the leakage hole induced a semicircular potential anomaly (ΔV = 70 mV). This spatial pattern aligns with the theoretical radial diffusion profile of a point-source electric field, confirming the establishment of a preferential conductive pathway within the leakage channel.

(2) Leakage Intensification Stage (t = 12–25 s):

Under sustained hydraulic head differentials, fine sand within the leakage channel was progressively displaced, reducing channel resistivity and accelerating seepage velocity. Consequently, the potential anomaly amplitude exhibited rapid amplification (ΔV_max_ = 113mVt(=25s); Fig 15b). This behavior corroborates the electrokinetic mechanism, where fluid flow generates measurable streaming potentials.

(3) Leakage Attenuation Stage (t = 26–90 s):

As the hydraulic head difference diminished to 40 cm by t = 80 s (Fig 15c), seepage deceleration led to a gradual decline in anomalous potential. The ΔV stabilized at approximately 85 mV, representing a 120% increase over initial values. This sustained anomaly enhancement is attributed to increased channel conductivity following water saturation, which amplified current density and thereby elevated the potential anomaly magnitude.

The experiments demonstrate a robust positive correlation between seepage velocity and potential anomaly intensity. During pit dewatering tests, localized hydraulic gradient surges in leakage zones generate pronounced seepage flows, significantly amplifying anomalous potential signals. The close agreement between experimental observations and numerical simulation results strongly validates the reliability of the computational model. So, these findings validate the theoretical framework for leakage localization based on seepage-electric field coupling. By monitoring spatiotemporal dynamics of dynamic potential anomalies, inverse identification of pit leakage pathways becomes feasible, offering a novel approach for real-time risk assessment in geotechnical engineering.

## 6 Conclusions

In this paper, the basic equations of the leakage electric field in pit pumping test are theoretically derived, and the three-dimensional orthogonal evolution of the pit leakage electric field in pit pumping test is realized by using the nonstructural finite element-infinite element method. A detection device of pit leakage electric field combined with pumping test is proposed. It is verified by numerical simulation and laboratory physical modeling experiments that the device enhances the potential anomaly signal in the leakage area and can locate the leakage channels quickly and effectively. The following conclusions are drawn:

(1) By leveraging the coupling characteristics between the leakage current field and the stable electric field, we derived equations for the electric fields generated by artificial current sources, the self-induced electric fields due to electrokinetic effects during pumping tests, and the electric fields associated with foundation pit leakage. These equations provide a comprehensive understanding of the mechanisms governing the formation of electric fields in foundation pits during pumping tests.
(2) The integration of the Foundation Pit Leakage Electric Field Detector with pumping tests enables rapid identification of potential leakage anomalies. During tests, self-potential in leakage areas increases the anomalous potential amplitude under a stable DC electric field. Key factors include power supply location, current magnitude, and receiver electrode placement. For effective exploration, both transmitter and receiver electrodes should be positioned within the aquifer to ensure proper current transmission and proximity to the pit structure, optimizing measurement accuracy.
(3) The validity of this paper’s method is verified by laboratory physical modeling tests, which confirm that there is a significant positive correlation between the seepage velocity and the strength of the potential anomaly. In the pit pumping test, the strong seepage flow in the leakage area due to the increase of local hydraulic gradient can make the abnormal potential signal in the leakage area significantly enhanced. This provides a theoretical basis for the development of leakage localization technology based on seepage-electric field coupling effect. By monitoring the spatial and temporal evolution characteristics of dynamic potential anomalies, the inverse identification of pit leakage potentials can be realized.

## Supporting information

S1 File Model data.zip. This is the forward modeling data used in Sections 3.2 “Simulation Results of the Seepage Field” and 3.3 “Simulation Results of the Leakage Electric Field” of the paper.
(ZIP)

S2 File 4.2.1 zip. This is the data used in Section 4.2.1 “Effect of emission current” of the paper.
(ZIP)

S3 File 4.2.2 zip. This is the data used in Section 4.2.2 “Effect of emitting positive electrode burial depth” of the paper.
(ZIP)

S4 File 4.2.3 zip. This is the data used in Section 4.2.3 “Effect of the distance of the transmitter positive pole from the foundation pit enclosure structure” of the paper.
(ZIP)

S5 File 4.2.4 zip. This is the data used in Section 4.2.4 “Effect of seepage velocity” of the paper.
(ZIP)

S6 File 4.3.1 zip. This is the data used in Section 4.3.1 “Effect of burial depth of the leakage pathway” of the paper.
(ZIP)

S7 File 4.3.2 zip. This is the data used in Section 4.3.2 “Effect of horizontal position of leakage paths” of the paper.
(ZIP)

S8 File 4.3.3 zip. This is the data used in Section 4.3.3 “Effect of the number of leakage paths” of the paper.
(ZIP)

S1 Table Experimental data.xlsx. This is the experimental data used in Section 5 “Laboratory Experiments” of the paper.
(XLSX)
